# Cardiomyopathy in Duchenne Muscular Dystrophy: A Single-Center Experience

**DOI:** 10.1016/j.jacasi.2022.01.002

**Published:** 2022-04-19

**Authors:** Karthikeyan Kummitha, Ramya Ramesh, Ann Agnes Mathew, Madhuri Maganthi, Saravanan Palaniappan

Duchenne muscular dystrophy (DMD) is the most common inherited neuromuscular disorder in boys leading to progressive muscle damage.^1^ Myocardial involvement in DMD is frequent and a major cause of death. Diagnosis and management of cardiomyopathy in DMD (DMD-CM) is an evolving science, and guidelines lag behind current evidence. Therefore, a regional expert committee was formed in the State of Karnataka, India which reviewed the current evidence and evolved an expert consensus statement that was implemented in our dedicated neuromuscular disease clinic in 2019. Here, we report the results of our experience of the diagnosis and management of DMD-CM in accordance with that consensus statement.

A retrospective analysis was performed of registry data of patients with genetically confirmed DMD seen between January 1, 2019, and December 31, 2021. The study protocol was approved by the Institutional Ethics Committee of Bangalore Baptist Hospital, Bangalore, India.

A diagnosis of DMD-CM was made if patients had any 1 of the following:

• Echocardiographic evidence of left ventricular (LV) dysfunction (defined as LV ejection fraction [EF] of <50% using Simpson’s biplane method of discs).
• Abnormality detected using speckle tracking echocardiography (STE) to assess longitudinal, radial, and circumferential regional deformation or myocardial strain reported as global longitudinal strain (GLS). A GLS value of <–18 was considered abnormal (GLS estimate was performed using a Philips Epiq 7 cardiac ultrasound machine with Automated Cardiac Motion Quantification software [Philips Q-LAB 10] and a locally optimized preset protocol.)
• Any presence of regional motion wall abnormalities on echocardiography.^2^^,^^3^ Data from cardiac magnetic resonance (CMR), when available, was used to corroborate echocardiography findings.

We also used the data from the registry to evaluate any association between genetic test results and clinical onset/severity of DMD-CM, along with an evaluation of our practice of treating patients with DMD-CM (Table 1).

A total of 108 patients with DMD were seen in the cardiomyopathy clinic during the study period. Our cohort had a greater proportion (68%) of patients who were at or younger than 10 years of age (>10 years: 35 [32%]; ≤10 years: 73 [68%]). Seven of 108 patients (6.4%) had an EF of <50%. Of the 101 with normal EF, 58 patients had abnormal GLS on STE. Of the 58 patients with abnormal GLS, 16 (23%) had CMR, of whom 10 (63%) showed evidence of myocardial fibrosis. Of the 108 patients, 95 (88%) had major rearrangements (deletions and duplications), and 13 (12%) had small sequence variant. Patients with major rearrangements had a greater incidence (65%) of DMD-CM compared to those with small sequence variant (33%). All patients younger than 10 years of age with DMD-CM had major rearrangements. There was no association between location or length of gene deletion and presence of DMD-CM. Of 65 patients with DMD-CM, 64 were on an angiotensin-converting enzyme (ACE) inhibitor/angiotensin receptor blocker (ARB). Those with lower EF and/or symptoms of heart failure were on additional medications as appropriate.

There are, as yet, no clear guidelines on the diagnosis and management of DMD-CM. The American Heart Association position statement on the management of neuromuscular diseases states that the average age for the development of abnormal LVEF is 14.3 years.^4^ However, with advancement in cardiac imaging, earlier identification of cardiac involvement has become possible.

In our study, we show that 65 of 108 (60%) patients had evidence of DMD-CM based on the estimate of GLS by STE, whereas conventional criteria of EF identified only 7 of 108 patients. Thus, the use of STE has identified an additional 58 patients, as shown in previous prospective studies.^5^ Of these patients, 35 (54%) were younger than 10 years of age. Almost all—except 1 patient—with abnormal EF were older than 10 years of age, indicating that an abnormal EF is a late finding and that by using this criterion, we tend to miss early features of DMD-CM.

All patients were considered for CMR but, because of issues such as young age of patients and cost concerns, we focused on CMR for patients with abnormal GLS. Thus, 16 of 65 patients had CMR, of whom 10 showed evidence of fibrosis.

Our data also suggest that genetic sequence variant patterns of gene deletion and duplication might be associated with greater risk of developing DMD-CM at an earlier age, but there was no relationship between the location or length of exon deletion and onset or severity of DMD-CM.

In our series, 64 of 65 patients with evidence of DMD-CM were on an ACEI inhibitor/ARB, indicating an early and proactive approach to the use of ACE inhibitor/ARB, in keeping with other similar reports.^6^^,^^7^

In this series, we report that myocardial injury occurs quite early in life and that genetic sequence variant, such as deletions and duplications, might predispose to such early onset. We also report that the early identification of such injury is feasible by estimating the GLS with STE. This led to use of ACE inhibitor/ARB early in the course of illness, which is thought to confer benefit by delaying the onset of overt heart failure and by slowing the progression of myocardial injury. This also facilitated focused use of CMR in a resource-poor setting.

**Table 1:** Imaging for DMD Cardiomyopathy

	Yes	No
All age groups		
EF < 50% (n = 108)	7 (7/108)	93 (101/108)
STE (n = 101)	57 (58/101)	43 (43/101)
CMR (n = 16)	63 (10/16)	37 (6/16)
Age < 5 y		
EF < 50% (n = 8)	0 (0/8)	100 (8/8)
STE (n = 7)	86 (6/7)	14 (1/7)
CMR (n = 0)	—	—
Age ≥ 5 y		
EF < 50% (n = 100)	7 (7/100)	93 (93/100)
STE (n = 94)	55 (52/94)	45 (42/94)
CMR (n = 16)	63 (10/16)	37 (6/16)
Age ≤ 10 y		
EF < 50% (n = 35)	1 (1/73)	99 (72/73)
STE (n = 72)	47 (34/72)	53 (38/72)
CMR (n = 3)	33 (1/3)	67 (2/3)
Age > 10 y		
EF < 50% (n = 35)	17 (6/35)	83 (29/35)
STE (n = 29)	83 (24/29)	17 (5/29)
CMR (n = 13)	69 (9/13)	31 (4/13)

Values are % (n/N).

CMR = cardiac magnetic resonance; DMD = Duchenne muscular dystrophy; EF = ejection fraction; STE = speckle tracking echocardiography.
